# Short-term reduction of ankle spasticity after surgical lengthening of the triceps surae in chronic post-stroke patients: a retrospective cohort study

**DOI:** 10.3389/fneur.2024.1342777

**Published:** 2024-03-18

**Authors:** Martina Galletti, Davide Mazzoli, Paolo Zerbinati, Chiara Rambelli, Giacomo Basini, Paolo Prati, Francesca Mascioli, Stefano Masiero, Andrea Merlo

**Affiliations:** ^1^Gait and Motion Analysis Laboratory, Sol et Salus Hospital, Torre Pedrera di Rimini, Italy; ^2^Neuro-Orthopedic Unit, Sol et Salus Hospital, Torre Pedrera di Rimini, Italy; ^3^Section of Rehabilitation, Department of Neuroscience, University of Padova, Padova, Italy

**Keywords:** stroke, equinus foot deformity, spasticity, neuro-orthopedic surgery, aponeurectomy, muscle lengthening, Modified Tardieu scale, triceps surae

## Abstract

**Introduction:**

In post-stroke patients with equinovarus foot deformity (EVFD), soft tissue rearrangements may contribute to muscle overactivity when a muscle is stretched or tension is applied. Therefore, we investigated the effects of surgically restoring the triceps surae (TS) length and lengthening ability on TS spasticity.

**Methods:**

This retrospective study included chronic post-stroke patients who underwent neuro-orthopedic surgery inclusive of TS lengthening. TS spasticity was measured using the Modified Tardieu Scale (MTS) before and 1 month after surgery, both with the knee extended (KE) and flexed (KF). MTS variations were analyzed using the Wilcoxon test. The time from stroke onset was compared between patients with and without post-surgical spasticity using the *t*-test. Statistical significance was set at 5%.

**Results:**

A total of 120 patients with EVFD, aged 57 (12) years, ranging from 1 to 36 years from stroke, were included in the study. The median MTS_KE score significantly decreased from 3 (range 0–4) to 2 (0–4) (*p* < 0.001) after surgery. The MTS score decreased by ≥1 point in more than half of the sample. Notably, 19 and 32 patients were completely relieved from spasticity (MTS = 0) in the KE and KF conditions, respectively. Post-surgical spasticity did not depend on the time since stroke onset (*p* = 0.560).

**Discussion:**

TS lengthening led to a short-term reduction of spasticity in 41% and 63% of chronic post-stroke patients in the gastro-soleus complex and soleus, respectively, with complete relief observed in 21% and 30% of the sample. Surgical lengthening can be considered an effective treatment that not only restores joint range of motion but also may reduce spasticity, even in chronic patients.

## 1 Introduction

Equinus and equinovarus foot deviation (EFVD) are the most frequent lower limb deformities found in stroke survivors (1). In individuals with EVFD, the foot and the ankle are deviated downward and rotated internally, and clawed toes may also be present (2). EFVD often affects the functional ability of standing and walking safely and the activities of daily life. This results in a serious impact on the patients’ quality of life and increases the caregivers’ burden.

EFVD may gradually occur in post-stroke patients as a consequence of muscle overactivity and muscle rheological modifications, influencing this process as in a vicious cycle. The first is characterized by the pathological onset of muscle overactivity in response to different stimuli with impaired de-recruitments after a muscle contraction (3). The latter is mainly due to prolonged immobilization with the ankle deviated toward plantarflexion (4).

Changes in soft tissues, such as increased stiffness, viscosity, and contractures, further contribute to muscle spasticity (5–7). During stretching, the pulling force is more efficiently transmitted to the spindles in a muscle with reduced compliance due to muscle shortening and changes in the rheological properties of tissues (8). According to this rationale, the recovery of muscle physiological length and extensibility could result in a reduction of spindle hyperactivation during muscle stretch and, possibly, a decrease of muscle spasticity.

EVFD treatments in post-stroke patients with triceps spasticity are manifold. These treatments include focal muscle blockage by botulinum toxin injections (9, 10) or chemical neurolysis with phenol or alcohol (11), physical therapy interventions (12), orthotic interventions (13, 14), and neuro-orthopedic surgery (15–18). Different surgical approaches have been developed targeting the calf muscles of patients with EVFD. These approaches include gastro-soleus complex recession (the Vulpius or Baker procedure), Achilles tendon lengthening (open-Z, percutaneous Hoke procedure, or White procedure), gastrocnemius aponeurosis lengthening (the Strayer procedure), gastrocnemius and soleus intramuscular lengthening (the Baumann procedure), and minimally invasive percutaneous fibrotomy. Surgical lengthening determines an immediate increase in length and compliance to lengthening of the treated muscle–tendon unit.

Two recent and independent studies reported a significant decrease in the triceps surae (TS) spasticity after surgical lengthening of the calf muscles in children with cerebral palsy (19, 20), along with the expected increase in ankle mobility. Among post-stroke patients, a reduction in the knee extensor spasticity was observed in 30 out of 52 subjects who underwent quadriceps femoris lengthening via percutaneous aponeurectomy (18). To date, no studies investigated the effect of surgical TS lengthening on TS spasticity in post-stroke patients with EVFD.

In this study, we analyzed the short-term variation in TS spasticity, measured by the Modified Tardieu Scale (MTS) (20), in a broad sample of post-stroke patients who underwent surgical EVFD correction including TS lengthening.

## 2 Methods

### 2.1 Study design and settings

This was an observational cohort study. Over the period between June 2012 and June 2020, we retrospectively analyzed the data from patients with chronic hemiplegia following stroke who had undergone neuro-orthopedic surgery to correct EFVD at our institution. Clinical and instrumental data were retrieved from the database of the Gait & Motion Analysis Laboratory at our institution. Patients were assessed both before and 1 month after surgery.

The significance of a short-term assessment is multifaceted. First, it allows us to identify changes in the patient’s walking pattern following the biomechanical modifications gained from surgery. Then, this new condition—i.e., joint range of motion and muscle activation patterns—is key to designing a tailored rehabilitation program. Finally, the assessment at the 1-month mark serves as the baseline reference for evaluating the effectiveness of the physiotherapy program and monitoring the patient’s recovery over time (21).

The manuscript was written according to the Strengthening the Reporting of Observational Studies in Epidemiology (STROBE) guidelines (22).

### 2.2 Participants

We included adult patients with: (a) left or right hemiparesis following an ischemic or hemorrhagic stroke (diagnosis confirmed by either computed tomographic scan/magnetic resonance imaging or clinical documentation); (b) chronic stroke (>12 months from the acute event); (c) EFD or EFVD; (d) first neuro-orthopedic surgery to correct EFD or EFVD deformity, inclusive of TS lengthening; (e) available MTS scores at the ankle both before and 1 month after surgery, and (f) available written and signed informed consent to personal data processing for research purposes. The exclusion criteria were as follows: (a) previous neurotomies or surgery to correct the EFD and (b) any treatment aimed at reducing spasticity in the prior 6 months.

This study was approved by the local ethics committee (CEIIAV Prot. 5953/2017 and 8484/2017).

### 2.3 Intervention

The intervention was carried out according to the TIDIER guidelines (23).

Patients underwent tailored neuro-orthopedic surgery, always inclusive of TS lengthening. The surgical procedure of choice was determined based on both clinical and instrumental gait assessments, including gait analysis and dynamic electromyography (7, 24, 25), conducted at the Gait and Motion Analysis Laboratory at our hospital.

The same surgeon (author PZ) with more than 20 years of experience performed all the interventions, ensuring consistency throughout the procedures. The EVFD correction involved lengthening of the Achilles tendon (the Hoke procedure), lengthening of the gastro-soleus complex (the Vulpius procedure), or other percutaneous fibrotomies. The Hoke procedure was used when severe contractures, typically ≥20 degrees of plantarflexion, were present. Otherwise, the Vulpius procedure was the standard approach. In a few patients, percutaneous fibrotomies were performed at the discretion of the surgeon. In some cases, correction of the supination and the varus component was achieved through a tuned combination of the following procedures: posterior tibial tendon lengthening; release of toe flexor tendons; split anterior tibialis transfer; anterior transfer of the flexor hallux longus; and extensor hallucis longus (EHL) transfer on the fourth metatarsal bone. The interventions to correct foot deviations in the frontal plane were designed and based on both the clinical assessment and the results of dynamic surface electromyography.

After surgery, all patients received a standardized intensive physiotherapy led on a 1:1 basis by a professional (16). This consisted of 24 sessions lasting 90 min, 6 days a week for 4 weeks. It included passive and active ankle mobilization, resistance and stretching exercises, and early gait training with a non-articulated ankle foot orthosis (16). Patients’ admission to our facility during the entire post-operative rehabilitation program ensured complete adherence to treatment.

### 2.4 Primary outcome

The primary outcome of this study was the variation in calf muscle spasticity at 1 month after EVFD surgery. Spasticity was assessed using the MTS score. This is a semi-quantitative, five-level ordinal scoring system based on the strength and duration of the stretch reflex. The following MTS formulation was used (26):

0—Absence of muscle reaction.

1—Weak resistance throughout the stretching movement, without a clear catch.

2—A clear catch, followed by release.

3—Fatigable clonus, < 10 s.

4—Infatigable clonus, >10 s.

5—Joint immobile.

Ankle passive range of motion was measured during slow-velocity stretching (V1), while muscle reaction was measured during passive stretching of the triceps surae at the fastest velocity (V3) (20). Assessments were performed with the patient lying supine on a bed, both with the knee extended (KE) and knee flexed (KF). All measurements of the participants were recorded by the same two experienced examiners to reduce potential bias.

### 2.5 Secondary outcomes

The secondary outcomes of this study included ankle range of motion, dorsiflexor muscle strength, and walking ability.

We measured both passive and active maximum dorsiflexion (pDF, aDF) with the KE and KF at 90 degrees, using a handheld goniometer. The manual muscle test of the Medical Research Council (MRC), with a score range of 0–5, was used to assess the tibialis anterior (TA), the extensor digitorum longus (EDL), and the EHL (27).

Mobility, walking disability, and limitations in social participation were measured using the Functional Ambulation Category (FAC, with a score range of 0–5) (28) and the Rivermead Mobility Index (RMI, with a score range of 0–15) (29). Gait speed (m/s) was derived from the instrumented gait analysis, which involved three walking trials on a 12 m-long walkway at a self-selected speed.

In addition, for each patient, we analyzed the pre- and post-surgical use of wheelchairs, orthoses, and walking aids such as canes or walkers.

The Patient’s Global Impression of Change (p-GIC, ranging from 1 to 7) score was collected after surgery (30).

### 2.6 Statistical analysis

Paired values, before and 1 month after surgery, were compared for all variables using the non-parametric Wilcoxon test (31) which was selected based on the preliminary analysis of data distributions (Shapiro–Wilk test). The Wilcoxon test was used to analyze the variations in paired variables. The non-parametric McNemar test was used to test for differences before and after surgery in dichotomous variables, such as the use of orthoses and walking aids (32). The presence of an association between post-operative spasticity (absent when MTS = 0, and present when MTS > 0) and the number of years since the lesion was assessed using the *t*-test.

Variations in TS spasticity were then analyzed at the single-subject level. Absolute frequencies of the patients with worsened, unchanged, and improved MTS scores were also determined (18). A reduction in the MTS score by ≥1 point was used to classify patients as improved (I). On the other hand, an increase of ≥1 point was used to classify patients as worsened (W). Patients were otherwise considered stable (S). In this study, the analysis of MTS scores was performed separately for patients with assessable pre-surgical spasticity (MTS ≤ 4) and those with non-assessable pre-surgical spasticity caused by complete retraction (MTS = 5).

The Wilcoxon test requires a sample of 39 patients to achieve an 80% power in the occurrence of data with a non-normal distribution and a medium effect size (*d* = 0.5) (33).

Statistical significance was set at 5% for all analyses.

## 3 Results

We screened records over an eight-year period, from June 2012 to June 2020. The data of 237 patients were available in our database, with 173 of them being stroke patients. Based on the eligibility criteria, 120 patients were included in the study (Refer the flowchart of patient selection in Supplementary file 1). Before surgery, MTS_KE was assessable in 89 patients, but was not assessable in 31 patients due to fixed contractures. Similarly, MTS_KF was assessable in 106 patients, but not assessable in 14 patients due to fixed contractures. Complete sample characteristics are presented in Table 1.

**Table 1:** Sample characteristics.

Parameter	Baseline characteristics
Sample size	120
Sex (F/M)	62/58
Age, years	57 (12); 21–80
Affected side (left/right)	72/48
Type of stroke (hemorrhagic/ischemic/n.a.)	44/69/7
Time from stroke, years	5.9 (6.2); 1–36
Time from surgery, years	5.8 (2.2); 2–10
Previous BoNT at the plantar flexors, y/n/n.a.	83/29/8
Use of myorelaxant drugs, y/n/n.a.	45/61/14
*Type of TS lengthening* Vulpius Hoke Percutaneous fibrotomies	90 26 4

The data are presented as counts, means (standard deviation), and ranges for numerical variables and as medians and ranges for ordinal variables.

### 3.1 Short-term effect of surgical muscle lengthening on spasticity

**Figure 1:**
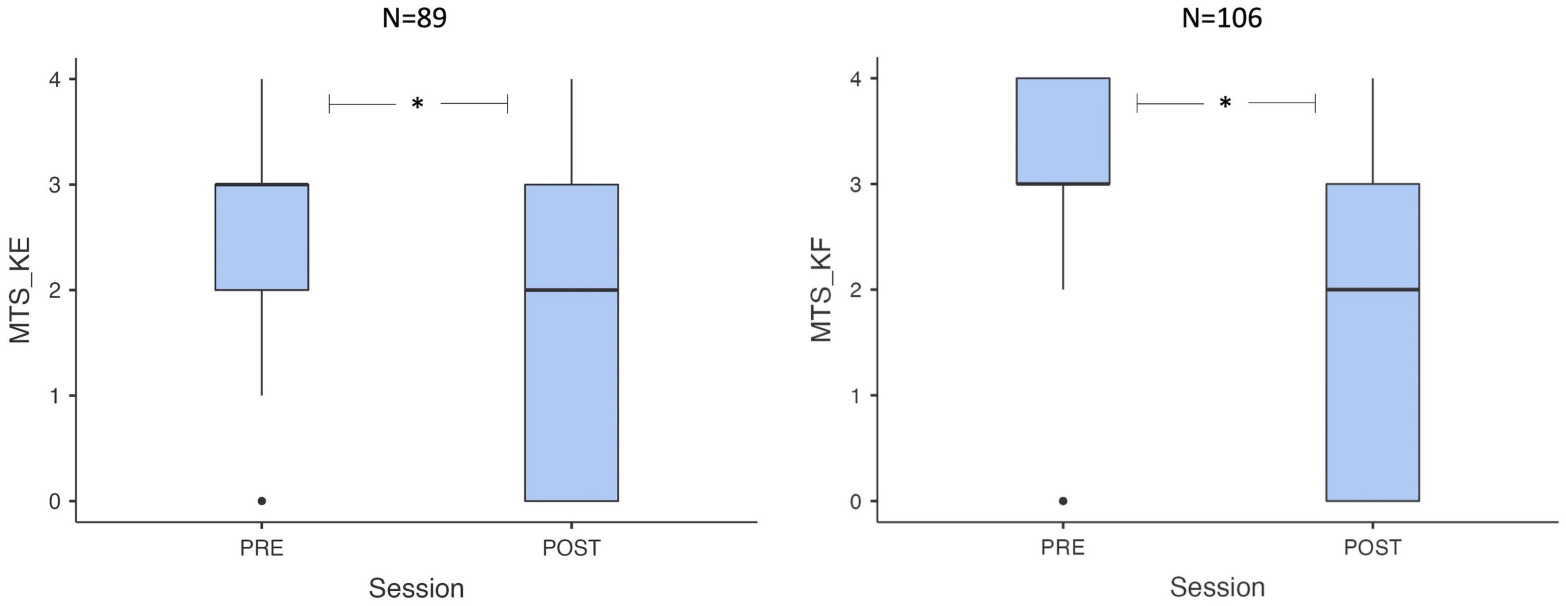
Box plots showing pre-post value distribution for MTS_KE e MTS_KF. MTS, Modified Tardieu Scale; KE, knee extended: KF, knee flexed.

The MTS_KE score significantly decreased at the 1-month mark after surgical lengthening (*p* < 0.001, Wilcoxon test), with a reduction in the median value from 3 to 2 (ranging from 0 to 4) in the 89 patients with assessable spasticity at the gastro-soleus complex. Similarly, the MTS_KF score decreased (*p* < 0.001, Wilcoxon test), with a reduction in the median value from 3 to 2 (range 0–4) at the 1-month mark in the 106 patients with assessable spasticity at the soleus, as shown in Figure 1. Complete results on TS spasticity variation at the 1-month mark after neuro-orthopedic surgery are presented in Table 2.

**Table 2:** Two-way table of MTS scores assessed with the knee extended and flexed at baseline and 1 month after surgery inclusive of TS lengthening.

	Ankle spasticity 1 month after surgery											
	Knee extended (MTS_KE)						Knee flexed (MTS_KE)					
		0 (*N* = 51)	1 (*N* = 2)	2 (*N* = 16)	3 (*N* = 42)	4 (*N* = 9)		0 (*N* = 54)	1 (*N* = 1)	2 (*N* = 22)	3 (*N* = 38)	4 (*N* = 5)
Ankle spasticity before surgery	0 (*N* = 19)	15	1	1	1	1	0 (*N* = 12)	11	0	0	1	0
	1 (*N* = 1)	1	0	0	0	0	1 (*N* = 0)	0	0	0	0	0
	2 (*N* = 10)	5	0	1	4	0	2 (*N* = 11)	7	0	2	2	0
	3 (*N* = 43)	11	0	8	22	2	3 (*N* = 55)	21	1	15	18	0
	4 (*N* = 16)	2	1	3	5	5	4 (*N* = 28)	4	0	3	16	5
	5 (*N* = 31)^1^	17	0	3	10	1	5 (*N* = 14)^1^	11	0	2	1	0

Light green highlights patients improved considering a minimal clinically important difference (MCID) of 1. Deep green highlights patients improved considering an MCID of 2. Light red highlights patients worsened considering an MCID of 1. Deep red highlights patients worsened considering an MCID of 2.

^1^Since pre-operative spasticity cannot be assessed in the presence of fixed contracture (5 = joint immobile), variations from 5 to lower values were not counted.

At the pre-surgical baseline assessment of the gastro-soleus complex, 70 patients had MTS_KE ≥ 1, 19 patients had no spasticity, and 31 patients were retracted (i.e., not assessable by MTS). At the 1-month mark, spasticity at the gastro-soleus complex improved by at least one point in 36 out of 70 (51%) patients, remained stable in 28 (40%), and worsened in 6 (9%) patients. It is worth mentioning that 19 out of 70 (27%) patients with pre-surgical MTS_KE ≥ 1 were completely relieved from spasticity at the 1-month mark, with MTS scores decreasing from 4 to 0 in 2 cases, 3 to 0 in 11 cases, 2 to 0 in 5 cases, and 1 to 0 in 15 cases. In 16 out of 59 (27%) patients, clonuses disappeared. In the 19 patients with MTS_KE = 0, spasticity arose after surgical lengthening in 4 cases, presenting weak resistance to stretching (1 case), a clear catch (1 case), fatiguing (1 case), and non-fatiguing (1 case) clonus. There were 31 patients with a gastro-soleus baseline contracture (i.e., non-assessable). Of these, 17 of them had no spasticity after surgery and 14 of them had MTS_KE scores ranging between 2 and 4, 1 month after surgery.

At the pre-surgical baseline assessment of the soleus, 94 patients had MTS_KF ≥ 1, 12 patients had no spasticity, and 14 patients were retracted (i.e., not assessable with MTS). At the 1-month mark, spasticity at the soleus improved by at least one point in 67 cases (71%), remained stable in 36 (38%) cases, and worsened in 3 (4%) cases. Furthermore, 32 (34%) subjects were completely relieved from spasticity, with MTS scores decreasing from 4 to 0 in 4 cases, 3 to 0 in 21 cases, and 2 to 0 in 7 cases at the 1-month mark. Clonuses were abolished in 40 out of 83 (48%) patients at the soleus. In the 14 patients with MTS_KF = 0, spasticity arose after surgical lengthening in three cases, presenting a clear catch (2 cases) and fatiguing clonus (1 case).

The suppression of spasticity (yes/no) at the gastro-soleus and the soleus after TS lengthening was not related to the time since stroke (*p* = 0.465, and *p* = 0.560, respectively), with MTS scores decreasing from 4 to 0 in both 2-year and 22-year chronic patients after the acute event.

### 3.2 Short-term effect of surgical muscle lengthening on ankle ROM, walking ability, and patient satisfaction

Baseline and post-surgery values for all clinical and functional variables collected in this study are presented in Table 3, along with the magnitude (effect size) and statistical significance (*p* value) of the pre–post variation.

**Table 3:** Baseline and 1-month follow-up values of the included sample (*N* = 120).

Parameter	Pre-surgical values	Post-surgical values	Effect size	*p*-value
Plantar flexors spasticity				
MTS_KE *N* = 31 not assessable due to retraction *N* = 89 assessable	- 3 (1); 0–4	- 2 (3); 0–4	- 0.658	- <0.001
MTS_KE spasticity angle, degrees (*N* = 70)	5 (15); 0–30	5 (15); 0–35	−0.091	0.596
MTS_KF *N* = 14 not assessable due to retraction *N* = 106 assessable	- 3 (1); 0–4	- 2 (3); 0–4	- 0.928	- <0.001
MTS_KF spasticity angle, degrees (*N* = 89)	10 (15); 0–40	10 (20); 0–30	0.327	0.020
Ankle range of motion, degrees				
Passive maximum DF_KE	−15 (15); −50–0	10 (5); −20–20	−1.000	<0.001
Passive maximum DF_KF	5 (15); −50–20	15 (10); −5–25	−0.990	<0.001
Active maximum DF_KE	−35 (20); −65–-5	−15 (25); −40–15	−0.985	<0.001
Active maximum DF_KF	−10 (16); −65–10	0 (15); −30–15	−0.913	<0.001
Dorsiflexors force				
MRC tibialis anterior	4- (3-); 0–5	4- (3-); 0–5	0.013	0.933
MRC extensor digitorum longus	1 (3); 0–5	1 (3); 0–5	0.146	0.350
MRC extensor hallucis longus	1 (4-); 0–5	1 (3); 0–5	0.310	0.042
Walking ability				
FAC	4 (2); 0–5	4 (1); 0–5	0.389	0.010
RMI	10 (4); 1–14	10 (6); 1–14	0.485	<0.001
Walking speed, m/s (*N* = 81)	0.33 (0.27); 0.08–0.88	0.32 (0.18); 0.07–0.88	0.209	0.148
Use of walking aids, *n* (%)	85 (71%)	88 (73%)	–	0.439
Use of orthosis, *n* (%)	69 (58%)	17 (14%)	–	<0.001
Use of wheelchair for outdoor mobility, *n* (%)	44 (36%)	47 (39%)	–	0.513

The data are presented as medians (IQR) and ranges for ordinal and numerical variables and as absolute and percentage frequencies for dichotomous variables.

n.a. not available; MTS, Modified Tardieu Scale; KE, knee extended: KF, knee flexed; MRC, Medical Research Council; DF, dorsiflexion; FAC, functional ambulation category; WHS, Walking Handicap Scale; RMI, Rivermead Mobility Index. The Wilcoxon test was used for comparing paired numeric and ordinal variables. The McNemar test was used for comparing paired proportions.

One month after surgery, ankle pDF was improved in both KE and KF conditions, increasing from −15° (range: −50°; 0°) to 10° (range: −20°; 20°) (*p* < 0.001) and from 5° (range: −50°; 20°) to 15° (range: −5°; 25°) (*p* < 0.001), respectively. Ankle aDF increased in both KE and KF conditions, varying from −35° (range: −65°; −5°) to −15° (range: −40°; 15°) (*p* < 0.001) and from −10° (range: −65°; 10°) to 0° (range: −30°; 15°) (*p* < 0.001), respectively.

Muscle strength did not change after 1 month (*p* > 0.05).

Walking ability and autonomy from caregivers slightly decreased after 1 month as assessed using FAC and RMI (*p* = 0.010, *p* < 0.001 respectively) (Refer Supplementary file 2 for details). Walking speed remained stable over time (*p* = 0.148).

Of the 69 patients who previously used lower limb orthoses, 56 dismissed them after neuro-orthopedic surgery (81%) showing a significant reduction (*p* < 0.001). Four patients started to use them. The remaining patients maintained their pre-surgery condition.

Patient satisfaction scores ranged from 1 to 6 on the p-GIC scale, with a median value of 2 (IQR = 1), meaning “much improved.”

## 4 Discussion

The study shows, for the first time in the literature, the short-term effects of TS surgical lengthening on TS spasticity in a sample of adult post-stroke patients.

The main finding of this study is that spasticity at the gastro-soleus complex and the soleus was reduced by surgical lengthening in 41% and 63% of the patients, respectively, with a significant median reduction in the MTS score of one point. Moreover, 32 patients were completely relieved from spasticity at the soleus (MTS_KF = 0), while 19 of them were relieved at the gastro-soleus complex (MTS_KE = 0) (Refer Table 2).

Spasticity refers to a disabling symptom affecting 7–46% of stroke patients. Studies have demonstrated that it significantly reduces the ability to actively participate in activities of daily life, affecting physical functioning and vitality, and causing physical and emotional limitations (34, 35). Thus, our results lay the foundation for a treatment with a substantial clinical connotation, as the reduction of spasticity may result in improved quality of life.

Neuro-orthopedic surgery, involving the release of muscles and related connective tissues, leads to a reduction in muscle passive tension and enables better compliance with stretching, and this is supposed to result in lower activity of neuromuscular spindles (4, 6, 8). Our results confirm that soft tissue passive modifications that develop in chronic post-stroke patients can contribute to exaggerated stretch reflexes and clonuses, at varying degrees among patients (8). On the other hand, patients who did not improve after surgery may have had more central-related overactivity components and may not have been sensitive to such intervention, mainly addressed to peripheral structures. Alternatively, an equal combination of both mechanisms may have been present, further increasing the complexity of clinical manifestations (36). As the knowledge of the mechanisms underlying this result is still to be developed, these hypotheses deserve to be examined in further studies, eventually inclusive of neurophysiological measures (e.g., the H/M ratio or the tonic stretch reflex threshold) (37, 38) or dynamic electromyography (7, 24, 39–42).

Another relevant finding of our study is the MTS scores obtained from patients not assessable at baseline, due to severe TS retraction that prevented the mobilization and stretching maneuvers. Most of them, after TS lengthening, obtained MTS scores of 0, presenting no sign of spasticity or clonus during fast stretching maneuvers (Refer Table 2). These findings further support the conclusions of the studies conducted by Gracies, Baude, and Trompetto (4, 6, 8, 36, 43) which highlighted the existence of different central and peripheral phenomena underlying EVFD that should be thoroughly evaluated so as to select the most appropriate treatment.

To our knowledge, there are only a few studies in the literature dealing with the effect of surgical muscle lengthening on spasticity. A case–control study observed the effects of quadriceps femoris aponeurectomy in adult stroke patients, registering comparable positive effects of neuro-orthopedic surgery on muscle spasticity as measured with MTS (18). Two studies found similar findings after lower limb neuro-orthopedic treatment in cerebral palsy children; however, the results were reported using the Modified Ashworth Scale scores (19, 44).

Shifting the focus on ankle DF, restoring the neutral position on the sagittal plane was achieved after surgery in our sample, with the ankle reaching an additional 10–15 degrees of dorsiflexion when passively mobilized (Refer Table 3). Moreover, when asked for aDF, patients could achieve the neutral position in the KF condition. If transferred to functional skills, this ability could ensure lower limb adequate clearance during the swing phase of the gait, thus reducing the risk of tripping or falling (45). It has been demonstrated that, during inertial tasks such as walking, a minimum muscle force is required by dorsiflexor muscles to reduce EFD during the swing, when no posterior brake is present (46). The recovery of ankle DF, along with the presence of both medial (TA) and lateral (EPA, EDL) dorsiflexor muscles balancing their action with no more posterior shortening of the TS, may have contributed to the disposal of lower limb orthoses in most of the patients who had previously used them (47). The use of orthoses for managing deformities may influence patients’ quality of life and their self-image (48, 49). In our study, 81% of the patients who used orthoses before surgery stopped using them after neuro-orthopedic surgery, due to the correction of the foot deformity. It is possible that the remarkable improvement perceived by the patients, as assessed using the p-GIC scale, is also related to the end of their use and the greater ease of walking (41). Walking ability decreased after 1 month. This was mainly due to the inability of some patients to perform the more complex tasks required by the scales (e.g., climbing stairs) considering the short recovery period after their recent surgery (Refer Supplementary file 2). On the other hand, walking speed remained unchanged. This was foreseeable as walking is a complex task requiring several skills that chronic stroke patients are not expected to recover within only 1 month. Longer follow-ups would have probably registered some improvements, as suggested by the current literature (50).

Regarding clinical practice, TS lengthening has several advantages. It is a simple, minimally invasive procedure and does not require high levels of specialization or complex procedures to be performed. It also involves a short recovery period for patients, with a low risk of side effects or adverse events, the possibility of weight-bearing on the operated limb as early as the following day after surgery, and wearing an ankle orthosis for 2–3 weeks, thereby allowing the early start of the rehabilitation program.

### 4.1 Sample characteristics and external validity of the results

In this study, we enrolled chronic patients who sustained a stroke (time from stroke 5.9 ± 6.2 years; range 1–36 years) with hemiparesis as the clinical outcome. The development of EVFD is one of the main consequences following both hemorrhagic and ischemic strokes, occurring within just a few months after the acute event up to several years, so our sample is truly representative of a large part of the stroke survivor population. As EVFD is the main deformity and one of the main causes for referral to treatment in stroke patients (17), this study has certainly addressed a relevant issue that deserves to be examined and studied in depth.

Our findings demonstrate the efficacy of neuro-orthopedic surgery in both 2-year and 22-year chronic patients, making it worthy of consideration in any stage of recovery. The efficacy of TS lengthening on ankle DF recovery and TS spasticity reduction in such a heterogeneous population in terms of chronicity and age, as preliminarily demonstrated in our findings, supports the external validity of our study.

### 4.2 Study limitations

The main limitation of our study is the lack of a control group, with a group of patients undergoing the same amount of physiotherapy but without undergoing surgery. As a result, we did not control for possible confounding factors. This could result in overestimating the positive effects of TS lengthening on TS spasticity. In the study conducted by Merlo and his colleagues on the effects on spasticity of percutaneous quadriceps lengthening, the authors considered a control group of patients who only underwent distal surgery (18). A similar approach was not possible in our study since all surgical patients recorded in our database had undergone surgery inclusive of TS lengthening. We attempted to compensate for this shortcoming by obtaining a large sample size. Future trials may compare the effects of surgical lengthening to other treatments aimed at reducing soft tissue stiffness, such as stretching, shock waves, or dry needling (12).

The absence of long-term data represents another limitation of the current study that limits the clinical applicability of our findings. In our study, the decision to include only short-term assessments was mainly driven by data availability constraints. Patients who seek treatment at our institution usually come from all over the country and do not come back after their first intensive rehabilitation period. It is, therefore, quite challenging to call them back for long-term follow-ups, which are usually carried out at their local institutions. Future studies should encompass clinical and electrophysiological measures of spasticity over several months after surgical muscle lengthening, assessing whether the patients remain stable or progressively worsen and possibly identifying both the positive and negative long-term predictive factors.

## 5 Conclusion

In the short term, TS lengthening reduced spasticity in approximately one out of two chronic post-stroke patients and abolished it in 20% and 25% of the subjects at the gastro-soleus and soleus, respectively.

Surgical TS lengthening can then be considered as an additional treatment of triceps spasticity in chronic post-stroke patients with a limitation in ankle range of motion.

Future studies should corroborate this finding, which is new in the literature, including follow-up assessments and electrophysiological measures of spasticity.

## Data availability statement

The raw data supporting the conclusions of this article will be made available by the authors, without undue reservation.

## Ethics statement

The study involving humans was approved by the local ethics committee (CEIIAV Prot. 5953/2017 and 8484/2017). The studies were conducted in accordance with the local legislation and institutional requirements. The participants provided their written informed consent to participate in this study.

## Author contributions

MG: Conceptualization, Writing – original draft, Data Curation. DM: Conceptualization, Methodology, Writing – review & editing. PZ: Writing – review & editing. CR: Data curation, Writing – review & editing. GB: Data curation, Writing – review & editing. PP: Data curation, Writing –review & editing. FM: Data curation, Writing – review & editing. SM: Writing – review & editing. AM: Conceptualization, Methodology, Writing – original draft, Writing – review & editing.
