# Supplementary material for: Short-term reduction of ankle spasticity after surgical lengthening of the triceps surae in chronic post-stroke patients: a retrospective cohort study
Source: Front Neurol. 2024 Mar 18;15:1342777. doi: 10.3389/fneur.2024.1342777 (PMC10984266; doi:10.3389/fneur.2024.1342777)
Supplement: Supplementary file 1 [file Data_Sheet_1.docx]

***Supplementary Material***

## Supplementary 1 – Flowchart of patient selection


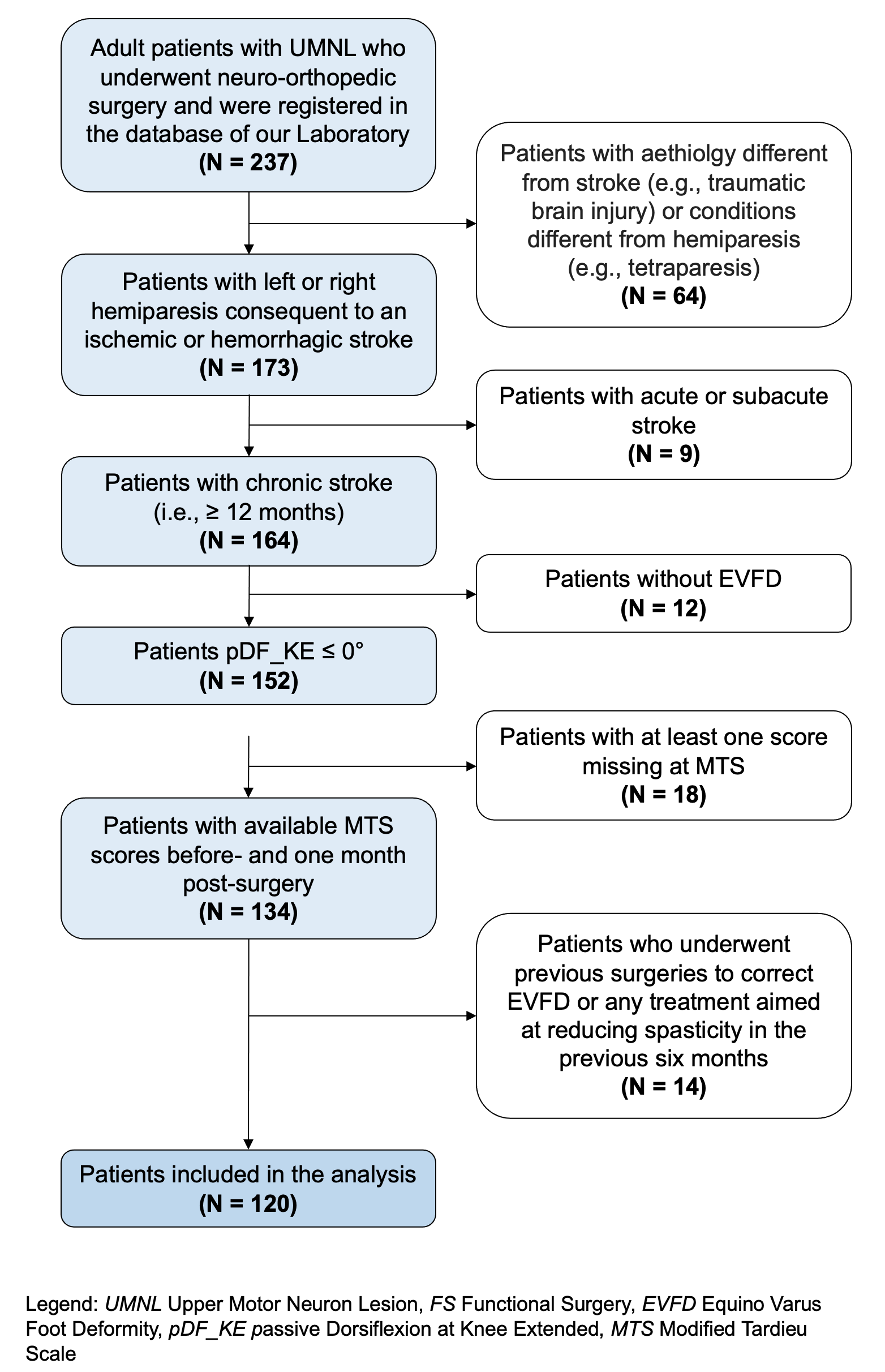


**Supplementary 1.** UMNL Upper Motor Neuron Lesion; EVFD Equino Varus Foot Deformity, pDF_KE passive Dorsiflexion at Knee Extended, MTS Modified Tardieu Scale

**Supplementary 2 - Box plots showing pre-post value distribution for the Functional Ambulation Category and Rivermead Mobility Index.**

|  |  |
| --- | --- |
| 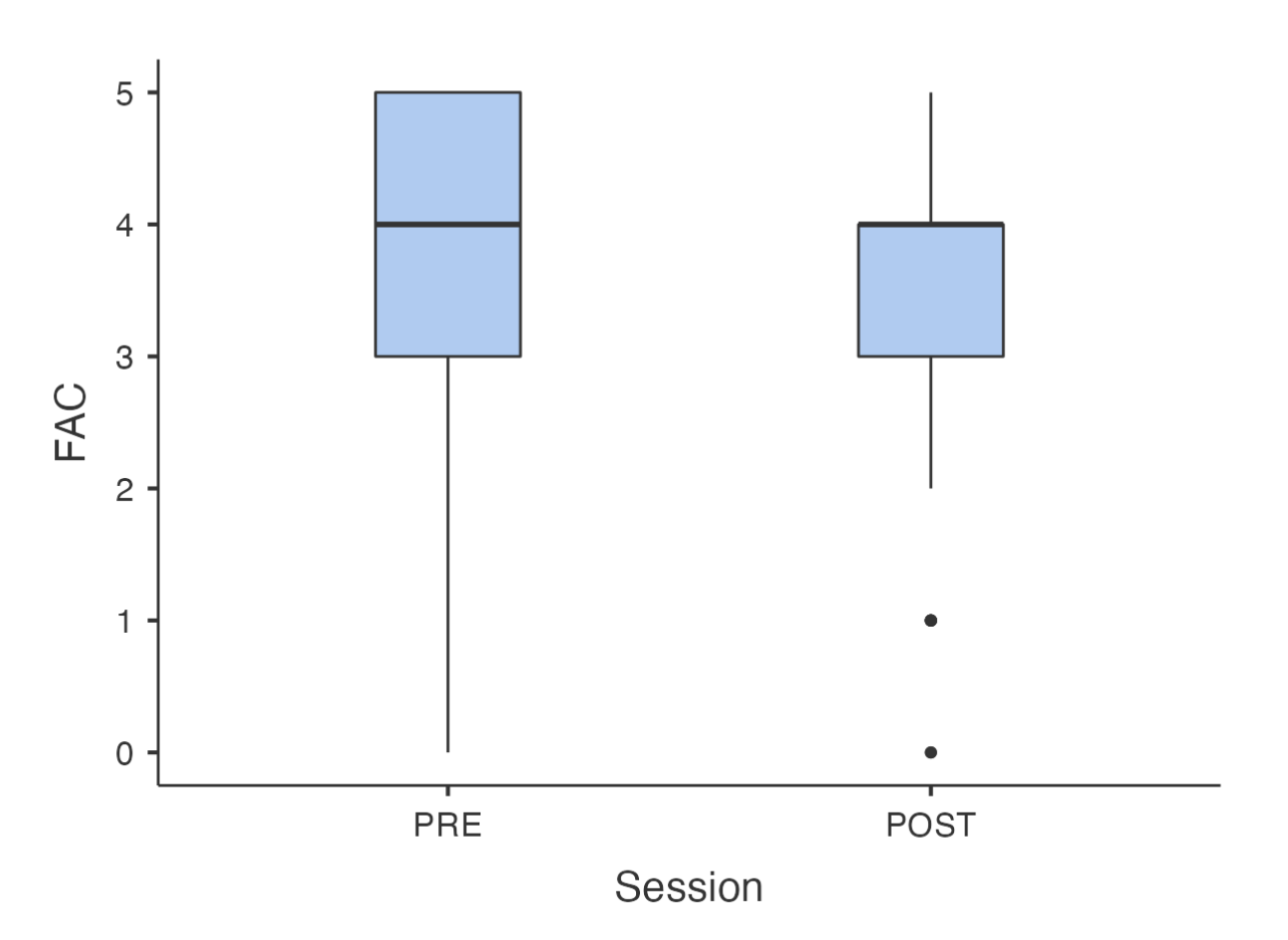 | 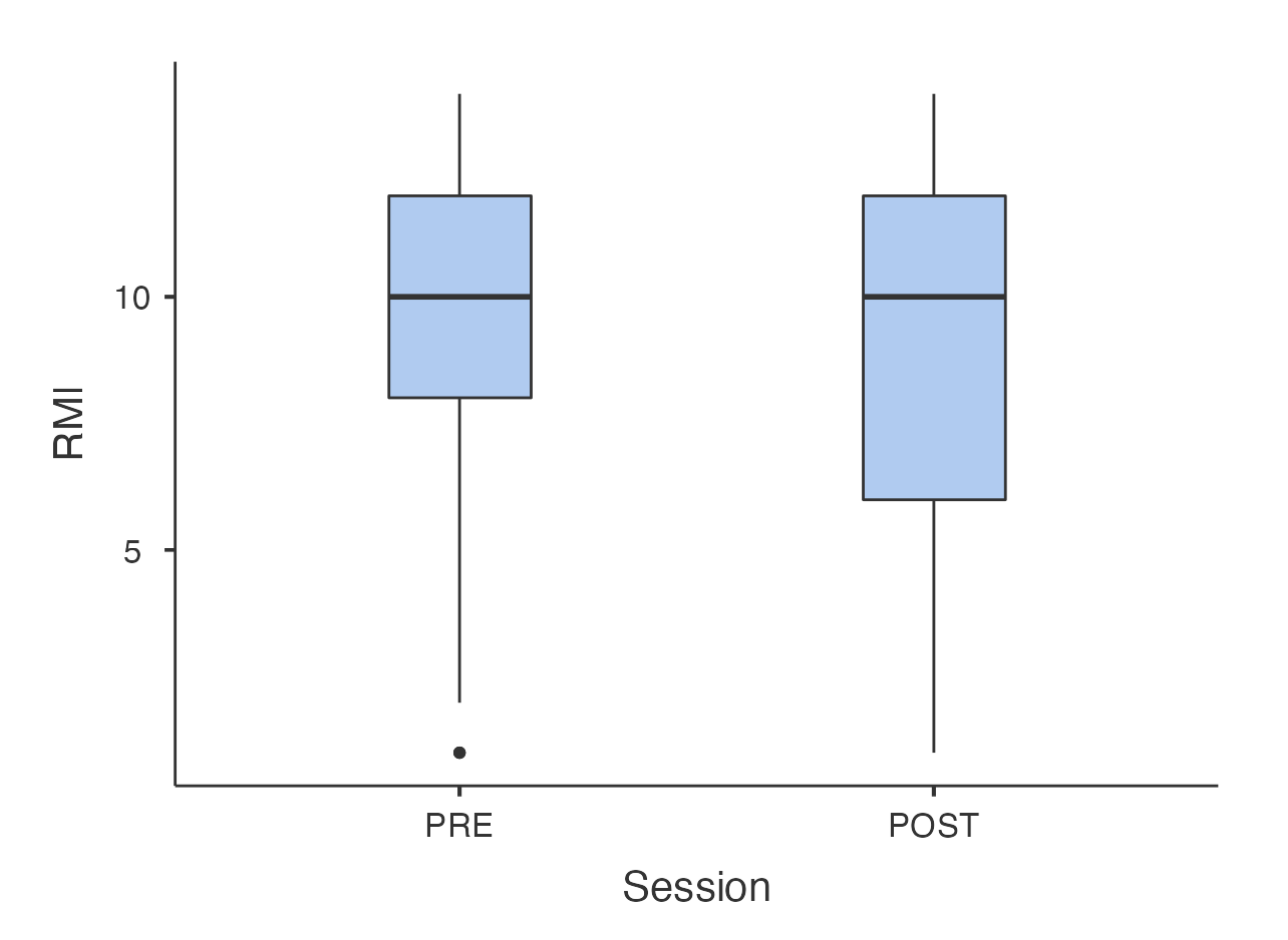 |

**Supplementary 2.** FAC Functional Ambulation Category; RMI Rivermead Mobility Index
